# A microbiome study reveals the potential relationship between the bacterial diversity of a gymnastics hall and human health

**DOI:** 10.1038/s41598-022-09355-x

**Published:** 2022-04-05

**Authors:** Z. Liang, C. B. Dong, H. Liang, Y. X. Zhen, R. L. Zhou, Y. F. Han, Z. Q. Liang

**Affiliations:** 1 Gymnastics Department, Chengdu Sport University, Chengdu, 610041 Sichuan China; 2 Institute of Fungus Resources, Department of Ecology, College of Life Sciences, Guizhou University, Guiyang, 550025 Guizhou China; 3 Physical Education College, Guizhou University, Guiyang, 550025 Guizhou China

**Keywords:** Microbiology, Bacteria, Metagenomics

## Abstract

Currently, investigations on the microbiota of sports centers and related facilities have been carried out in some countries, which showed that *Microsporum gypseum*, *Trichophyton mentagrophytes* and *T. tonsurans* are important dermatoprotofungi. In China, some research on athletes and sports equipment between the fungal community and public health has made some interesting achievements. However, the bacterial group among them has not been reported. Therefore, The aim of this study was to uncover (I) gymnastic equipment is there potential pathogenic factors and (ii) is there any difference in the biomarker of bacterial in different types of gymnastic room? The samples were collected from the gymnastics halls of one university in western China and main sports equipment, including gymnastics carpets, moving barres, hoops and balls, as well as wall bars, parallel bars and horizontal bars. The 16S rDNA of all the samples was sequenced, and the analyses were performed using FaproTax, Bug base function prediction and Line Discriminant Analysis (LDA) Effect Size. A 16S rDNA sequence analysis revealed abundant bacterial species biodiversity on gymnasts and apparatuses from two gymnastics halls at a university in western China. An analysis using the FaproTax and Bugbase functional prediction platforms showed that there were some opportunistic pathogens on the athletes and equipment from the Rhythmic Gymnastics (RG) and Artistic Gymnastics (AG) halls, such as *Staphylococcus* and Corynebacteiaceae. Infectious agents associated with cancer induction and development, such as Ruminococcaceae, Veillonellaceae and Moraxellaceae, as well as microbial toxin producers with a potential impact on human health, were also detected. According to a line discriminant analysis (LDA effect size), the bacterial biomarker groups of the two gymnasiums were different at the phylum-genus level: for RG, Erysipelatoclostridium, Lachnospiraceae and Bacteroidales, while for AG, Rhizobiales. Based on the results of the investigation, we suggest that more comprehensive consideration should be given to indoor microbial biodiversity and related public health problems in school gymnasiums.

## Introduction

At present, 55% of the world's population has moved from rural to urban areas^1^. The referenced study observed that people living in towns and cities interact with microbes in markedly different ways from those living in rural areas. Microorganisms in a built-up environment have been considered a possible source of infection^2,3^. Obviously, living in a city has a significant impact on human health, and the mechanism of that impact varies greatly^4^.

Urban transportation systems, including subways and buses, are utilized daily by billions of urban residents. Travelers, in turn, move around the city carrying their symbiotic microbes, adding to the complexity of urban microbes. At present, studies on urban environmental microbiota in transportation systems^5^, hospitals^6,7^, soil^8^, and sewage^9^ have been performed. For example, Danko et al.^4^ found 10,928 viruses, 1302 bacteria and 2 archaea in a survey of 4728 metagenomic samples from mass transit systems. All of these studies show that public urban public spaces contain abundant microbes. However, there are many other indoor public places where people often gather, such as karaoke halls, airports and bus stations, as well as sports venues and other facilities, for which research reports are uncommon.

In recent years, the correlation between the diversity, relative abundance, and function of microbiota and respiratory diseases in the campus environment (including dormitories and classrooms) has been studied in some countries^10–13^. These studies observed that the highest abundance of Actinobacteria, Gammaproteobacteria and Alphaproteobacteria was in urban schools, and Actinobacteria and Cyanobacteria were more abundant in rural schools^11,13^. Fu et al.^12^ also found that *Izhakiella* and *Robinsoniella* were positively correlated with the severity of asthma in a study of classroom floor dust in junior middle schools in Malaysia.

By the 2020s, some countries had also carried out microbiota studies on swimming pools, showers, saunas, wardrobes, wrestling mats, gymnastics carpets and pillowcases in sport centers. In most cases, dermatoprotofungi such as *Microsporum gypseum*, *Trichophyton mentagrophytes* and *T. tonsurans* were found^14^. Sports venues and corresponding equipment have become carriers or storehouses of pathogenic fungi^15^. Liang et al.^16–18^ from China conducted the first investigation based on the rDNA-ITS sequence to study the structure, dynamics and potential public health problems of fungi on some participants and sports equipment of school soccer teams and rhythmic gymnastics in China. However, there are few reports on the bacterial microbiome structure of such sports venues and its relevance to public health.

In this study, athletes and the main apparatuses in two gymnasiums from a university in western China were selected as the research subjects. Based on the 16S rDNA sequence, the bacterial community structure and function will be analyzed to uncover that gymnastic equipment was there potential pathogenic factors and was there any difference in the biomarker of bacterial in different types of gymnastic room.

## Materials and methods

### Test items and sampling methods

#### Rhythmic gymnastics group (RG)

Thirty female RG students at a university in western China were used in this study (age: 20.70 years; height: 165.20 cm; weight: 52.96 kg; training period: 7.16 years).

#### Tested items

Rhythmic gymnast palms (n = 30 persons), one rhythmic gymnastics carpet (13 × 13 m^2^), 3 barres (length: 4 m/barre), rhythmic gymnastics hoops (n = 30) and balls (n = 30).

#### Sampling method

##### Athlete

A small amount of sterile water was poured into a sterile medical bag to moisten the gauze. The tester wore disposable gloves (replaced for each sampled subject), wiped both of the subject’s palms 4–5 times with gauze after exercise, and then immediately put the gauze into a sterile plastic bag.

##### Sport equipment

The gymnastics carpet was sampled after training. The carpet was divided into 9 rectangles (diagonal: 30 cm × 30 cm) with wide adhesive tape. Three pieces of gauze were used to sample each rectangle. A gloved tester wiped each rectangle with wet gauze 4–5 times. The gauze was put into a sterile plastic bag. To compensate for insufficient biological samples obtained by wiping the carpet, some of the fibers on the surface of the carpet were cut as a sample at any three places in each sampling rectangle area.

##### RG apparatus

The hoop, ball and barre were sampled after gymnast training. A gloved sampler wiped the hoops and balls’ surface 2–3 times with wet gauze; each barre was wrapped with two pieces of wet gauze and wiped 2–3 times. After all the samples were collected, they were mixed into 5 pooled samples.

##### RG sample code

Carpet (aa), barre (ab), hoop (ac), ball (ad), palm (ae).

#### Artistic gymnastics group (AG)

Thirty-seven AG students at a university in Western China were included in the study, including 12 boys and 25 girls (age: 20.81 years; height: 165.97 cm; weight: 55.88 kg; training period: 4.57 years).

#### Tested items

Artistic Gymnast’s palms (37 persons), one gymnastics carpet (14 × 14 m^2^), 3 sets of gymnastics wall bars, 10 bars per set (length: 77 cm/bar), 3 pairs of horizontal bars (length: 23.7 cm/bar) and 4 pairs of parallel bars (length: 35 cm/bar).

#### Sampling method

##### Athletes

The same as the sampling method for rhythmic gymnasts.

##### Sports equipment

One gymnastics carpet was sampled after training. The carpet was divided into 9 rectangles (diagonal: 47 cm × 47 cm) with wide adhesive tape. Three pieces of gauze were used to sample each rectangle. A gloved tester wiped each rectangle with wet gauze 4–5 times. The gauze was put into a sterile plastic bag. To compensate for insufficient biological samples obtained by wiping the carpet, some of the fibers on the surface of the carpet were cut as a sample at any three places in each sampling rectangle area.

##### Gymnastic equipment

Samples were collected from the wall bars, parallel bars and horizontal bars after training. A gloved sampler whose gloves were replaced for each sampled piece of equipment wrapped each wall bar with two pieces of wet gauze and wiped the surface 2–3 times. The same method as for the wall bars was used to sample the parallel bars and horizontal bars. After all samples were collected, they were mixed into 5 pooled sample bags.

##### AG sample code

Carpet (ba), wall bars (bb), horizontal bar (bc), parallel bars (bd), palm (be).

### DNA extraction, amplification and sequencing

#### DNA extraction and PCR amplification

Total DNA was extracted using a FastDNA Spin Kit for Soil (MP, USA) according to the manufacturer’s instructions. DNA concentration and purity were detected using a NanoDrop2000, in which nucleic acid was selected after placing the 2 μL DNA sample, and the DNA extraction quality was detected by 1% agarose gel electrophoresis. The PCR experiment used 338F (5′-ACTCCTACGGGAGGCAGCAGCAG-3′) and 806R (5′-GGACTach VGGGTWTCTAat-3′) primers for PCR amplification of the v3–V4 variable region. The amplification procedure was as follows: predenaturation for 3 min at 95 °C, 27 cycles (95 cyclic denaturation for 30 s, 55 s annealing for 30 s, 72 for extended 30 s), and a maximum extension of 10 min (PCR: ABI GeneAmp 9700). The amplification system was 20 μL, 4 μL 5*FastPfu buffer, 2 μL 2.5 mm dNTPs, 0.8 μL primer (5 μM), and 0.4 μL FastPfu polymerase. Briefly, 10 ng DNA template. PCR products were recovered using a 2% agarose gel, purified using the AxyPrep DNA Gel Extraction Kit (Axygen Biosciences, Union City, CA, USA), eluted with 10 × TE buffer, and detected by 2% agarose electrophoresis. Quantification was performed using QuantiFluor uantiPromega, USA). PE 2*300 libraries were constructed from the purified amplified fragments according to the Illumina MiSeq platform (Illumina, San Diego, USA) standard operating procedures.

#### Data quality control

fastp (https://github.com/OpenGene/fastp, version 0.20.0) software was used for quality control of the original sequencing sequence, and FLASH (http://www.cbcb.umd.edu/software/flash, Version 1.2.7) software splicing was employed. For OTU sequence clustering, UPARSE software (http://drive5.com/uparse/, version 7.1) was used with a similarity of 97%, and chimeras were eliminated. Species classification annotation was performed using the RDP classifier (http://rdp.cme.msu.edu/, Version 2.2), and the comparison threshold was set to 70% for the Silva 16S rRNA database (V138).

### Data processing

Functional prediction. FAPROTAX is a functional prediction software based on 16S sequencing. It integrates multiple prokaryotic functional databases of published culturable literature. The database contains 7600 functional information for over 4600 species, including 80 functional groups including potential pathogenesis, methanogenesis, fermentation, and so on. When using, the annotated OTU table in Greengenes or Silva database is read, and the annotated OTU information is compared with the species information in the database through Python program, and the results of predicting various functions are output^18^. BugBase is another tool for predicting the phenotypic function of the 16S microbiome. Based on OTU tables and Mapping files, BugBase uses existing databases, annotations and frameworks to compare and measure a large amount of information, providing users with organism-level related phenotypic prediction of the microbiome^19^. Including Pathogenic Potential, Biofilm Forming, Gram Positive and Gram Negative and so on. BugBase also provides grouped statistics and visualization. BugBase can be used as a web application (http://bugbase.cs.umn.edu) to use, also can be downloaded for free (https://github.com/knights-lab/BugBase).

Bar chart of microbial composition and relative abundance of samples, Venn and KRONA Annotation, FaproTax and Bugbase function prediction analysis as well as LefSe analysis (http://huttenhower.sph.harvard.edulefse/) were performed using BMKCloud (http://www.biocloud.net/). Specifically, for species analysis with significant differences (known as biomarker analysis) between groups, the LefSe was used to estimate the impact of the abundance of each component (species) on the differential effect. The LDA score for significant differences was set to 3.0 and p < 0.05.

The Ethics committee of Chengdu Sport University approved the study protocol. The study was conducted in accordance with relevant guidelines and regulations.

### Informed consent

Informed consent was obtained from all individual participants included in the study.

## Results and analysis

### The bacterial community of gymnasts and commonly used instruments has abundant species diversity

**Figure 1:**
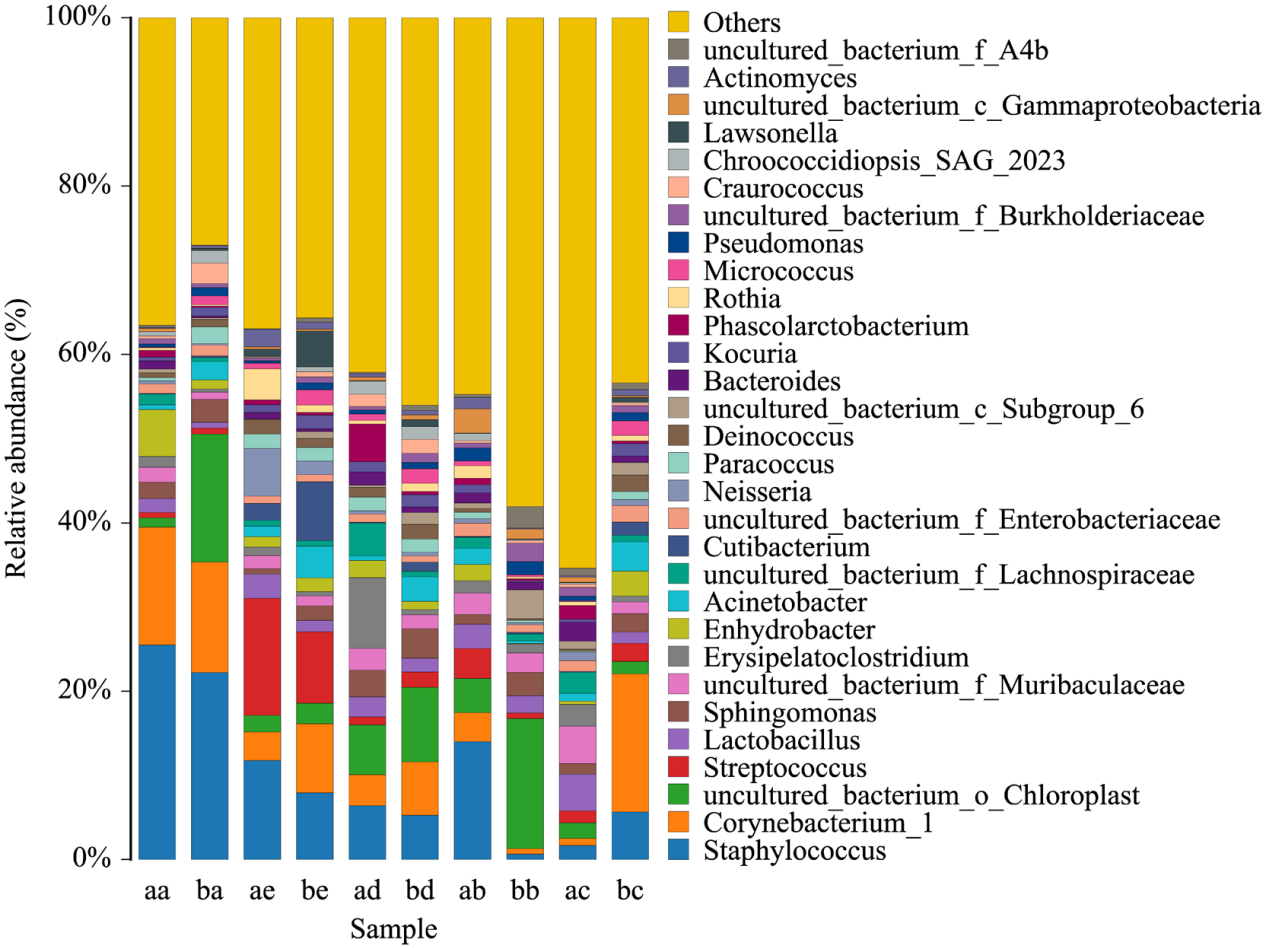
Bacterial composition and relative abundance of main apparatus in two gyms (Top 30). a/RG Gym: aa (carpet), ab (barre), ac (hoop), ad (ball), ae (palm). b/AG Gym: ba (carpet), bb (wall bars), bc (horizontal bar), bd (parallel bars), be (palm).

A total of 1926 OTUs were obtained by 623,404 clustering effective sequences, and these OTUs were annotated as 847 species of microorganisms belonging to 41 phyla, 99 classes, 225 orders, 374 families, and 746 genera of archaea and bacteria. These data indicate a rich diversity of bacteria on athletes' palms and the commonly used gymnastic apparatus (Fig. 1, Table 1). Some of these groups, such as Muribaculaceae, are anaerobic bacteria that specifically inhabit the gut. The abundance of these bacteria correlates with animal longevity^20,21^. *Lactobacillus* is the main probiotic in the human stomach and small intestine. Of these, a mixture of *Lactobacillus acidophilus* and *Bifidobacterium* sp. is good for regulating gastrointestinal dysfunction. *Sphingomonas* has multiple ecological functions ranging from remediation of environmental pollution to the production of highly beneficial plant hormones^22^. Other taxa, such as the order chloroplasts in Archaea and Lachnospiraceae, also have various ecological functions and application prospects^23,24^.

**Table 1:** The relative abundance (%) of some dominant groups in each sample.

Genus	aa	ab	ac	ad	ae	ba	bb	bc	bd	be
*Staphylococcus*	25.4	14.0	1.6	6.4	11.7	22.2	0.6	5.6	5.2	7.9
*Corynebacterium*	14.0	3.4	0.8	3.6	3.3	13.1	0.6	16.4	6.3	8.2
*Enhydrobacter*	5.5	1.9	0.3	2.0	1.2	1.0	0	2.9	0.9	1.6
*Sphingomonas*	1.9	1.1	1.2	3.1	0.6	2.7	2.7	2.1	3.5	1.7
Unc. F. Muribaculaceae	1.7	2.5	4.4	2.5	1.5	0.8	2.2	1.4	1.6	1.1
*Lactobacillus*	1.6	2.9	4.3	2.3	2.8	0.6	2.0	1.3	1.6	1.3
*Erysipelatoclostridium*	1.2	1.4	2.5	8.4	0.9	0.3	1.0	0.6	0.6	0.5
Unc. F. Lachnospiraceae	1.2	1.2	2.5	3.8	0.7	0.4	0.8	0.7	0.6	0.6
Unc. O. Chloroplast	1.1	4.0	1.8	5.9	1.9	15.1	15.4	1.4	8.8	2.4
Unc. F. Enterobacteriaceae	1.1	1.5	1.3	0.9	0.8	1.3	0.9	1.9	0.7	0.8

In the RG hall, the dominant bacteria on the carpet (aa) was *Staphylococcus*, with a relative abundance of 25%, followed by *Corynebacterium* (14%) and *Enhydrobacter* (5%) (Fig. 1). The bacterial composition of the AG hall carpet (ba) was similar to that of the carpet (aa), and the dominant groups were as follows: *Staphylococcus* 22%, a species of Cyanobacteria belonging to the order chloroplast 15%, *Corynebacterium* 13%, *Craurococcus* 2%, and *Acinetobacter* 2%.

On the carpet, except for a chloroplast (15%) in the AG hall, opportunistic pathogenic bacteria, *Staphylococcus* and *Corynebacterium* were predominant. Other equipment (except the wall bars [bb]) also generally contained *Staphylococcus* (including STRAIN S31), *Corynebacterium*, *Acinetobacter*, *Streptococcus*, Lachnospiraceae and an unidentified genus of Enterobacteriaceae.

### Opportunistic pathogenic factors were found in two gymnasiums and the main apparatus

**Figure 2:**
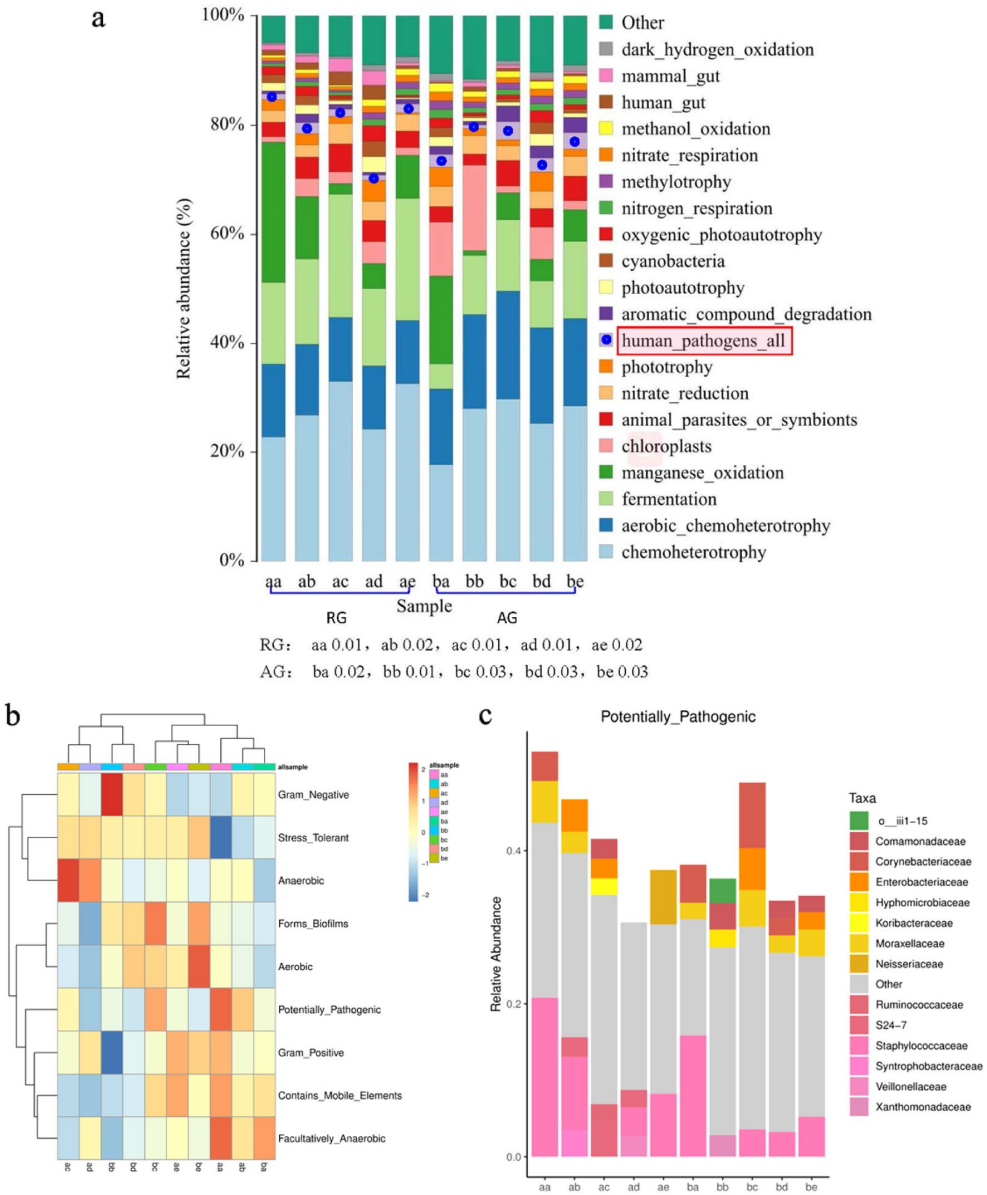
Bar chart of FaproTax function prediction of microbiome in two gyms, (**a**) (TOP 20 in functional abundance; P: 0.05). (**b**) Phenotypic functional maps of the microbiome of the two gyms predicted by BugBase algorithm; (**c**) opportunistic pathogens or dominant microbiota associated with cancer displayed at the family-level.

To confirm the existence of opportunistic pathogens in all analyzed bacterial groups, the FaproTax and Bugbase functional prediction platforms were used to predict the potential pathogens of humans in all samples from the two gyms (Fig. 2a–c). There were some differences in the relative abundance and opportunistic pathogen taxa. The overall trend indicated that the relative abundance in the AG hall was higher than that in the RG hall.

BugBase phenotypic function prediction also confirmed the existence of potential human pathogens in the two gyms. Visualizations (Fig. 2c) further showed that the specific taxa were Veillonellaceae, Ruminococcaceae, Moraxellaceae, Lachnospiraceae, Propionibacteriaceae and *Staphylococcus*. *Comamonas*, *Klebsiella*, *Rothia*, *Acinetobacter*, and *Micrococcus* are cancer-related microbial factors^25^.

### Shared bacterial groups and biomarker groups in different gymnasiums

**Figure 3:**
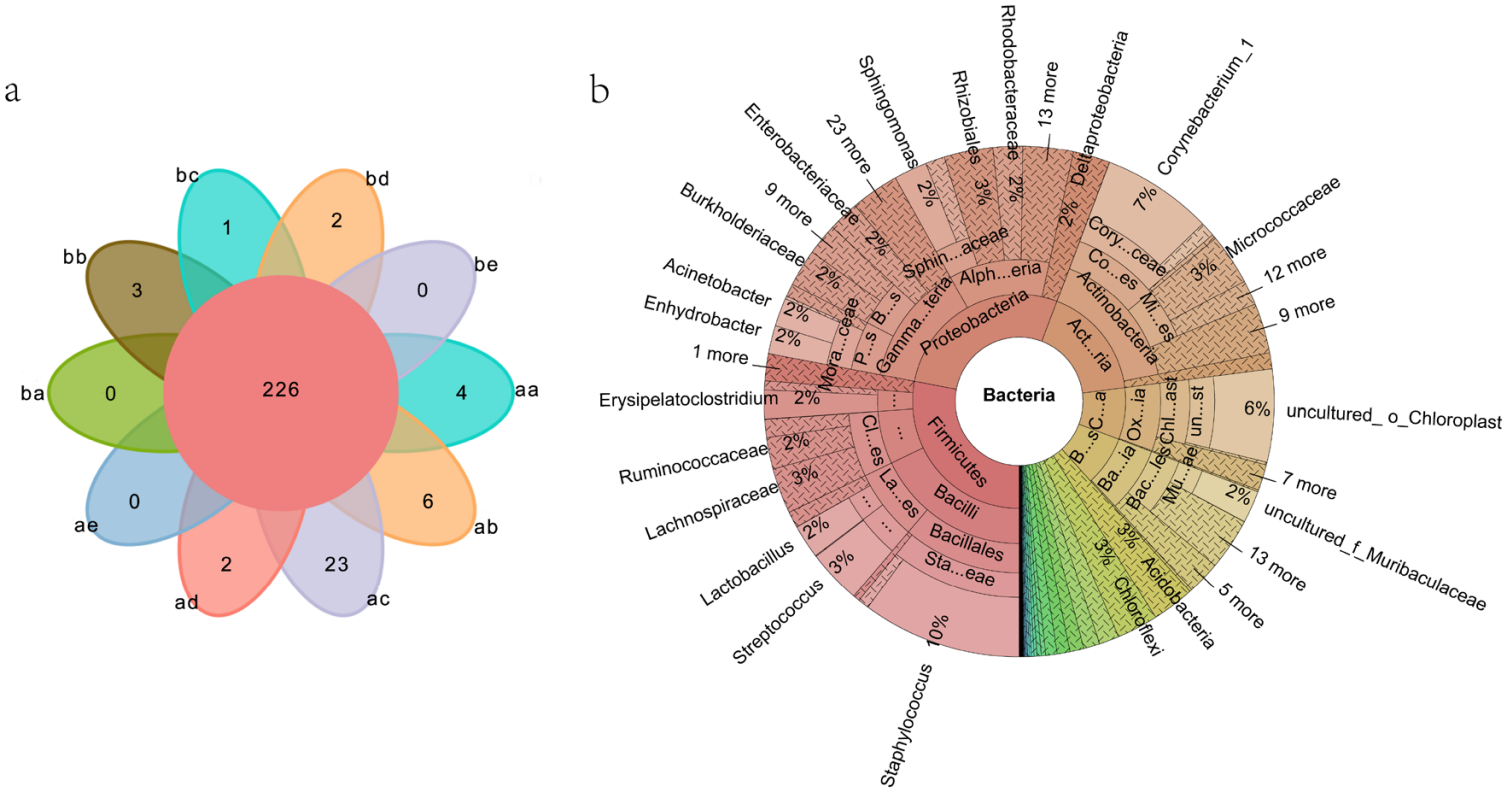
Venn distribution of a genus-level for all sample taxa. b KRONA species annotation map of all samples: RG/aa (carpet), ab (barre), ac (hoop), ad (ball), ae (palm); AG/ba (carpet), bb (wall bars), bc (horizontal bar), bd (parallel bars), be (palm).

A Venn diagram analysis showed that there were 226 taxa in all samples at the genus level, accounting for 30.3% of the total 746 genera (Fig. 3a). A KRONA species note diagram is further shown in detail (Fig. 3b). The relative abundance of *Staphylococcus*, *Corynebacterium* and *Enhydrobacter* in the two gymnastic halls was 5–25%. In addition, other groups with a relative abundance of 2% were Class Chloroflexi, Rhizobiales, Micrococcales, Muribaculaceae, Ruminococcaceae, Burkholderiaceae, Enterobacteriaceae, and *Lactobacillus*. *Acinetobacter*, *Acidobacteria*, *Oxyphotobacteria*. Chloroflexi is a typical member of the anaerobic photosynthetic taxa, mostly from hot spring-associated environments^26^.

**Figure 4:**
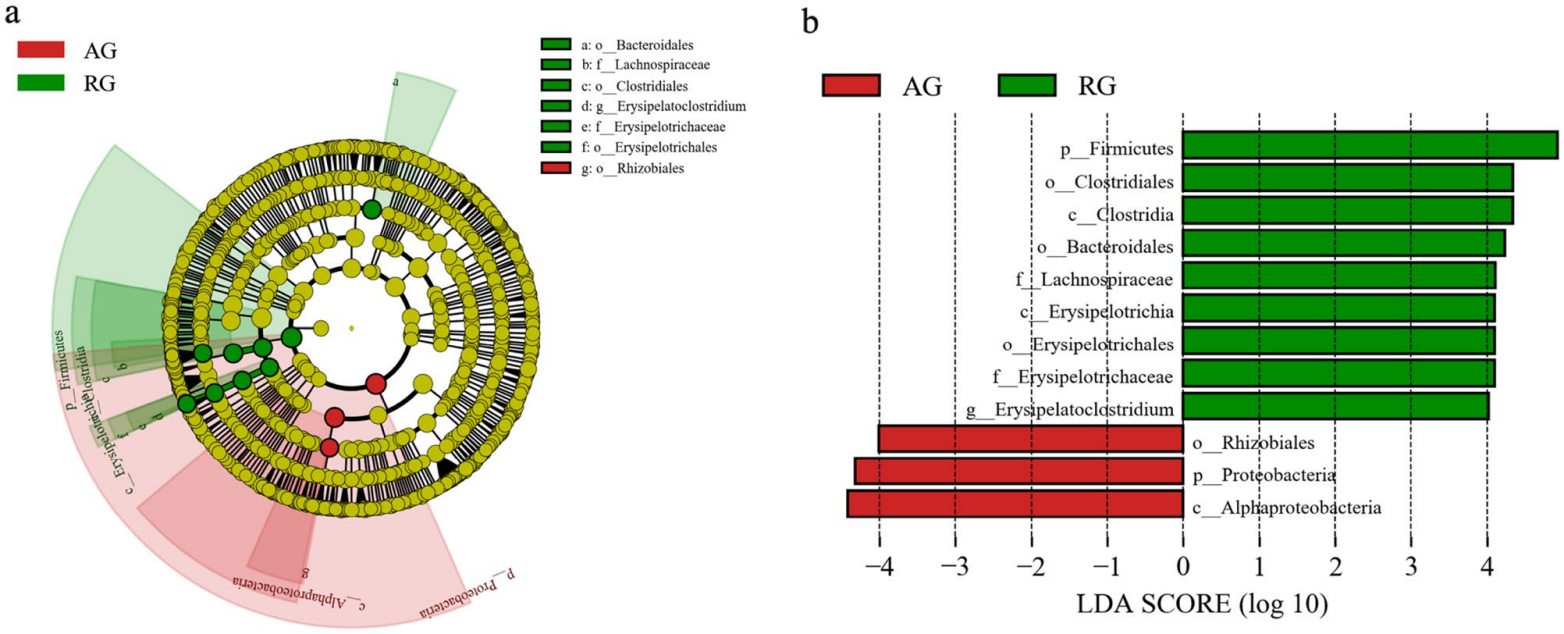
LEfse analysis of bacteria characterictic taxa in two gyms. (**a**) (from phylum to genus) cladogram; (**b**) bar diagram. The bacterial biomarker enriched in AG (red) in cladogram (**a**) is Rhizobiales. Similarly, as shown in the cladogram, the bacterial biomarkers of RG (green) in column figure (**b**) are *Erysipelatoclostridium*, and Lachnospiraceae and Bacteroidales. The node size in FIG.4.a represents the abundance of the group. b Histogram shows LDA score > 3 and p < 0.05.

Line discriminant analysis (LDA effect size) can identify biomarkers with significant differences between different groups. Figure 4a and b show the different microbial groups in the two gyms. In the AG hall (in red), the biomarker microorganism is Rhizobiales, while in the RG hall (in green), the biomarker microorganisms are *Erysipelatoclostridium* and *Lachnospiraceae*. *E. ramosum*, a species in the genus *Erysipelatoclostridium*, is a normal member of the human gut microbiota and only causes invasive infections in rare cases^27^. The members of Lachnospiraceae are the core taxa of the intestinal tract and have a high species abundance and relative abundance in the life cycle of the host. Although they are the main producers of short-chain fatty acids, different taxa are also associated with different enteral and extracteral diseases^24^. The AG hall had only one biomarker microbial group, Rhizobiales. A member of this order, Agrobacterium, can be used in genetic engineering. Genera associated with human and animal diseases include *Bartonella*, *Brucella*, *Pedomicrobium* and *Xanthobacter*. The first two genera were not found in our current survey.

## Discussion

The skin is home to millions of bacteria, fungi and viruses. In a survey of healthy adults, Byrd et al.^28^ found that on drier parts of the hand, such as the palm and forearm, the main bacteria were *Staphylococcus*, *Streptococcus*, *Corynebacterium* and *Propionibacterium* (*Cutibacterium*). On the more moist antecubital fossa and popliteal fossa, *Corynebacterium*, *Enhydrobacter*, *Propionibacterium* and *Staphylococcus* were found. For the lipid-based cheek and retroauricular crease, *Corynebacterium*, *Propionibacterium*, *Staphylococcus* and *Streptococcus* were dominant. The main bacteria on the feet are *Corynebacterium* and *Staphylococcus*. Kim et al.^29^ studied the surface microbes of different populations in South Korea and found that *Enhydrobacter* was dominant in the skin microbiome of the elderly, while *Lawsonella* was more prevalent in a younger group. *Staphylococcus* and *Corynebacterium* were dominant in males, while *Lactobacillus* was dominant in females.

**Figure 5:**
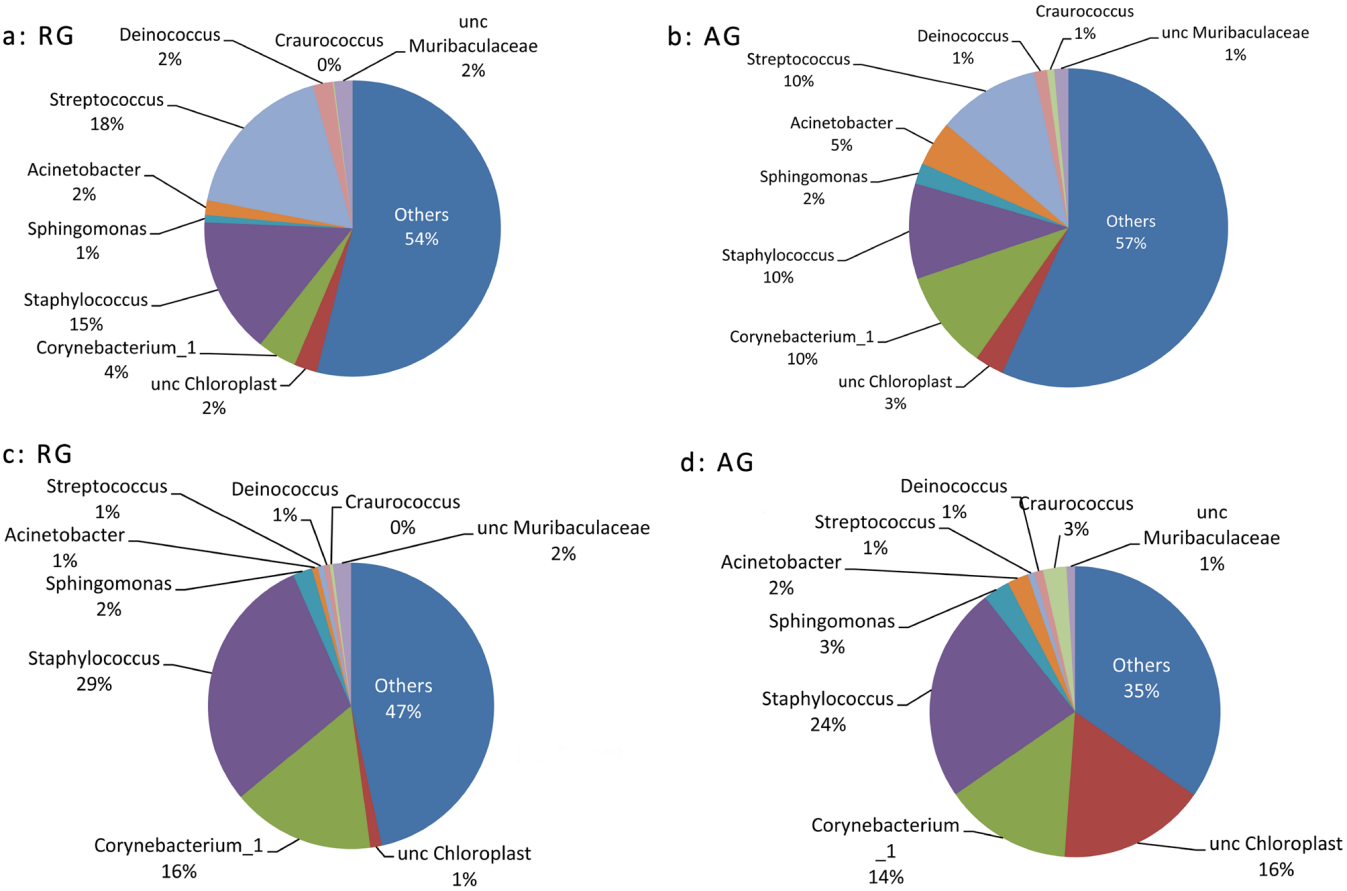
Composition of dominant taxon (top 10 taxa) of palms and carpets from RG and AG group. (**a**) palm of RG athlete, (**b**) palm of AG athlete, (**c**) RG carpet, (**d**) AG carpet.

In our study (Fig. 5), the dominant taxa in the palm front of 9 female gymnasts (RG) and male and female gymnasts (AG), in addition to *chloroplasts*, were *Streptococcus*, *Staphylococcus*, *Corynebacterium* and *Cutibacterium*, which was basically consistent with the report of Byrd et al.^28^. RG athletes have *Lactobacillus*, which is consistent with the report of Kim et al.^29^. AG, which contains a combination of male and female team members, contains more specific groups, including *Lawsonella*, *Acinetobacter*, *Micrococcus, Sphingomonas*, Order Chloroplas and *Kocuria*. The reason may be related to the physiology of male and female athletes and this AG openness to the outside world. *Acinetobacter*, for example, often comes from contaminated dairy products, raw fruits and vegetables.

In the two carpets of GR and AR that had more contact with athletes' hands and feet, the dominant microbiota similar to hands, including *Staphylococcus*. *Corynebacterium* and *Enhydrobacter* have increased, while *Streptococcus* has decreased (Fig. 5c,d). The hydrophilic bacteria *Enhydrobacter* are more abundant. The skin microbiome composition of the dominant types of bacteria is considerably stable; the diversity of the gym microbiome is closely related to the human body surface microbiome.

Cyanobacteria are widely found in seawater, fresh water, soil and the bark of some plants. Some of these cyanobacteria can produce harmful toxins, such as microcystins (characteristic hepatotoxins), nodularins and cylindrospermopsin. Anatoxin A and saxitoxins are neurotoxins, and their formation shows no strain specificity^30^. Common toxin-producing algae include *Microcystis* spp., *Anabaena* spp., *Aphanizomenon*, *Planktothrix* and *Oscillatoria*^29^. Radkova et al.^31^ reported that *Microcystis* produced microcystin in shallow water in Bulgaria, which is the cyanobacterial toxin found with the highest frequency; it has a wide distribution and high toxicity in cyanobacterial water blooms. Archaea have been detected on human skin, and they mainly belong to *Methanobacteriota* and *Halobacteriota*, which have relatively low abundance. Umbach et al.^32^ evaluated the relative abundance and taxonomic distribution of archaea on human skin based on 16S rRNA gene sequence data. Their results showed that only 5.9% of the 1,688 skin samples contained archaea. The dominant bacteria on computer keyboards in contact with finger skin are members of Nitrososphaeria. Our study found some rare groups of Cyanobacteria, such as Chroococcidiopsaceae, Calothrix, *Microcoleus* PC-7113, *Scytonema* UTEX 2349, Tychonema CCAP-459-11 and Nostocales and *Afrocarpus*, *Nannochloropsis*, and *Trachydiscus*. On the hands and carpets of RG and AG athletes. These taxa were not consistent with the groups reported by Svirčev et al.^33^ and Umbach et al.^32^. In addition to the differences in environmental conditions of sampling sites, an important reason for such inconsistency was that Svirčev et al.^33^ and Umbach et al.^32^ found that the reported microbes were common taxa with high relative abundance, while we found that the reported microbes were all with relative abundance < 0.001 rare taxon. The prediction based on the FaproTax and Bugbase functional platforms (Fig. 2) reveals that there are some opportunistic human pathogens in the gymnasium stadium. The opportunistic pathogens detected were *Lawsonella* spp. and *Corynebacterium pseudodiphthericum*. Streptococcae are widely found in nature and in human and animal feces, and most of them do not cause disease. We found that the relative abundance St. mutans was extremely low, but it is a key species in the caries process^34^. *Acinetobacter baumannii* contaminates dairy products, fruits and vegetables and increases the risk of infection in immunocompromised individuals and young children^35^. Some microorganisms are not directly opportunistic agents but are carcinogenic agents^36^. Yu et al.^37^ confirmed that *Fusobacterium nucleatum* and *Peptostreptococcus stomatis* are related to colorectal cancer. Several other species, including *Parvimonas micra* and *Solobacterium moorei*, have also been significantly associated with colorectal cancer. Some of the other microbes that we found by using functional prediction, such as Neisseriaceae, Ruminococcaceae, Veillonella and Moraxella, are also associated with the occurrence and development of cancer^25^. The multifaceted biodiversity of the microbiome on gymnasts, gyms and apparatuses should be fully understood and recognized by managers, teachers and athletes. In daily activities, participants should pay attention to the cultivation of personal and public health habits and regularly clean and disinfect equipment and instruments used. Some researchers and administrators have proposed their inclusion in health education programs and curricula^38,39^. Some schools have even set up ultraviolet sterilization lamps in gymnastic halls to improve the protection of athletes. These ideas and methods are worth learning from school administrators.

## Conclusions

Based on rDNA-16S sequencing, the results of this study showed that gymnasts and commonly used equipment had abundant species diversity, and the potential pathogenic factors involved not only opportunistic pathogens but also microbial infectious factors related to the occurrence and development of cancer. Some rare species of cyanobacteria merit more attention, such as *Calothrix*, *Microcoleus*, *Tychonema*, *Affrocarpus* and *Trachydiscus*, which were found in our survey. Whether these cyanobacteria produce toxins harmful to humans is still a concern.
